# Rehabilitation of a Patient With Bickerstaff Brainstem Encephalitis Presenting With Ataxia

**DOI:** 10.7759/cureus.80864

**Published:** 2025-03-20

**Authors:** Isao Uno

**Affiliations:** 1 Rehabilitation Medicine, Sakura Juji Fukuoka Hospital, Fukuoka, JPN

**Keywords:** ataxia, bickerstaff brainstem encephalitis, postural balance, rehabilitation, social participation

## Abstract

Bickerstaff brainstem encephalitis (BBE) is a rare autoimmune disorder characterized by oculomotor disturbances, ataxia, and impaired consciousness, likely caused by brainstem inflammation. Although acute immunotherapy, including corticosteroids, plasma exchange, and intravenous immunoglobulin, appears to effectively mitigate the inflammatory process, residual neurological deficits often persist, necessitating individualized rehabilitation strategies.

We report the case of a 40-year-old male with BBE who presented with severe impaired consciousness, ataxia, and diplopia following flu-like symptoms. Despite initial immunotherapeutic interventions, the patient continued to experience significant functional limitations, as evidenced by a Functional Independence Measure (FIM) score of 78, a Berg Balance Scale (BBS) score of 22, and a Scale for the Assessment and Rating of Ataxia (SARA) score of 20 upon admission to the rehabilitation ward. An intensive, stepwise, and individualized rehabilitation program was implemented. The protocol initially emphasized static and dynamic balance exercises on stable surfaces, trunk and limb strengthening, and environmental modifications to address diplopia. From the second week, task-specific gait training, including applied stepping, stair climbing, and oculomotor exercises, was introduced. After the third week, the regimen progressed to outdoor ambulation and activities simulating real-life tasks to facilitate social reintegration and occupational readiness. Following 41 days of rehabilitation, marked improvements were observed. The patient’s FIM score increased from 78 to 121, BBS improved from 22 to 56, and SARA score decreased from 20 to 3, indicating significant amelioration of ataxia. Enhanced performance on the Timed Up and Go (TUG) test and the 10-meter walk test, as well as an increased 6-minute walking distance, reflected improved balance, mobility, and endurance. Additionally, the patient’s diplopia improved, enabling safe ambulation and the successful resumption of leisure activities. This case underscores the pivotal role of an individualized, timely rehabilitation program in facilitating functional recovery in patients with BBE. Task-specific training and sensorimotor integration strategies appear to promote neural plasticity and improve motor and oculomotor functions. Further research is warranted to develop and validate evidence-based rehabilitation protocols tailored to BBE and similar autoimmune neurological disorders.

## Introduction

Bickerstaff brainstem encephalitis (BBE) is a rare autoimmune disorder characterized by ocular motility disturbances, ataxia, and altered consciousness. Its etiology is closely linked to molecular mimicry mechanisms associated with anti-GQ1b antibodies [1]. Epidemiologically, BBE is considered a rare disorder, with an estimated incidence of approximately 0.05-0.1 cases per 100,000 population per year [2]. Although precise figures vary due to differences in diagnostic criteria and reporting practices, BBE is generally less common than related conditions such as Guillain-Barré syndrome (GBS) and Miller-Fisher syndrome (MFS). A comprehensive understanding of its epidemiology is essential for appreciating the rarity of the condition and the consequent challenges in developing disease-specific treatment and rehabilitation protocols.

For clarity, 'molecular mimicry' refers to the phenomenon whereby antigens derived from pathogens share structural similarities with neural components, thereby triggering an autoimmune response. In this context, anti-GQ1b antibodies are believed to mediate BBE pathogenesis, distinguishing it from conditions such as MFS and GBS, which typically present with less pronounced brainstem involvement. BBE shares pathophysiological features with Miller-Fisher syndrome (MFS) and Guillain-Barré syndrome (GBS), often presenting with similar cranial nerve involvement and peripheral nervous system manifestations [3]. However, BBE typically exhibits more pronounced brainstem-related symptoms, such as impaired consciousness, and is generally more severe than other anti-GQ1b antibody syndromes. Although acute treatment with high-dose corticosteroids, plasma exchange, and intravenous immunoglobulin (IVIg) may ameliorate the clinical course, several studies have reported that residual neurological deficits, including sensory-motor impairments, compromised motor coordination, and difficulties with activities of daily living, persist in a proportion of patients [2]. These lingering sequelae underscore the need for effective and targeted rehabilitation interventions.

Despite the well-documented benefits of rehabilitation in various neurological disorders, there is a dearth of published reports describing rehabilitation protocols specifically tailored to the complex clinical presentation of BBE. However, rehabilitation strategies that adequately address the unique challenges posed by severe brainstem involvement, such as impaired consciousness and ocular motor dysfunction, remain underdeveloped, potentially impacting long-term functional recovery. For example, structured, task-oriented programs have proven effective in improving postural stability, reducing falls, and enhancing overall functional mobility in degenerative conditions such as spinocerebellar ataxia and multiple system atrophy. Although Bickerstaff brainstem encephalitis is an autoimmune inflammatory disease, it also presents with significant postural instability and impaired motor coordination. Thus, despite the differences in etiology, these rehabilitation strategies may be applicable to BBE, as they target common functional deficits. Future studies are warranted to directly assess their efficacy in autoimmune inflammatory conditions [4]. While a comprehensive rehabilitation approach addressing both central and peripheral deficits may be necessary for patients with BBE, current clinical protocols are largely adapted from those designed for other ataxic and autoimmune neurological disorders. To date, evidence-based guidelines specific to BBE remain scarce, necessitating reliance on experiences with similar conditions when formulating rehabilitation programs.

In light of these gaps, there is a pressing need for individualized rehabilitation approaches specifically designed for the distinctive clinical features of BBE. In this report, I present a case of BBE in which a comprehensive and progressive rehabilitation program effectively addressed persistent neurological sequelae, primarily severe ataxia, following acute immunotherapy. Interventions focused on task-specific exercises, balance training, and social reintegration yielded significant improvements in gait and functional independence. This case highlights the potential of well-structured rehabilitation to optimize functional recovery in patients with BBE-related ataxia and emphasizes the need for the development of disease-specific rehabilitation guidelines.

## Case presentation

A 40-year-old male with no significant past medical history presented with symptoms consistent with BBE. Employed as a researcher, he led an active lifestyle that included frequent outdoor activities and extensive use of public transportation. His activities of daily living (ADLs) were fully independent, and he regularly engaged in leisure activities with his family. Approximately 20 days prior to admission, he developed flu‐like symptoms after caring for his son, who had an upper respiratory tract infection. Specifically, the patient experienced fever, cough, and generalized malaise, which likely contributed to the rapid deterioration in his neurological status. Shortly thereafter, his level of consciousness deteriorated rapidly, necessitating emergency transport to a tertiary care hospital.

Upon admission, his Japan Coma Scale (JCS) score was 100, indicating severe impairment of consciousness. These clinical manifestations, including ataxia, diplopia, and altered consciousness, were consistent with the established diagnostic criteria for BBE, and the combination of ocular motor disturbances with central nervous system impairment was particularly valuable in differentiating BBE from conditions such as Miller-Fisher syndrome. His infectious workup was negative, supporting a non‐infectious etiology. The diagnosis of BBE was established based on the presence of central nervous system dysfunction, early ataxia, and oculomotor disturbances.

High‐dose intravenous methylprednisolone pulse therapy was initiated on day 3 after onset of symptoms, followed by oral prednisolone (30 mg/day). Due to the limited improvement observed following initial immunotherapy, plasma exchange was initiated to further suppress the inflammatory response, thereby contributing to the subsequent improvement in both consciousness and ocular motor function. On day 32, a 5‐day course of intravenous immunoglobulin (IVIg) therapy was administered, leading to further stabilization. On day 49, he was admitted to our rehabilitation ward.

Clinical findings

At the time of admission to the rehabilitation ward, his Functional Independence Measure (FIM) score was 78 (45 for motor function and 33 for cognitive function). His Berg Balance Scale (BBS) score was 22, reflecting a high risk of falls, and his Scale for the Assessment and Rating of Ataxia (SARA) score was 20, indicative of severe ataxia. Muscle testing demonstrated preserved upper extremity strength, with grip strength measurements of 23.1 kg on the right and 25.1 kg on the left (manual muscle testing [MMT] grade 5/5); however, lower extremity strength was mildly diminished bilaterally (MMT grade 4/5). Cognitive function was within normal limits, as evidenced by a Mini-Mental State Examination (MMSE) score of 30, a Trail Making Test B (TMT-B) completion time of 98 seconds, and a Frontal Assessment Battery (FAB) score of 17. Additionally, the patient exhibited diplopia, which impaired his ability to perform visual tasks and ambulate safely. He expressed a strong desire to return to work and to engage in leisure activities with his family, such as playing catch with his son (Table 1).

**Table 1:** Rehabilitation assessment FIM: Functional Independence Measure. BBS: Berg Balance Scale. SARA: Scale for the Assessment and Rating of Ataxia. MMT: manual muscle testing. MMSE: Mini-Mental State Examination. TMT-B: Trail Making Test B. FAB: Frontal Assessment Battery. TUG: Timed Up and Go. The FIM score reflects the patient’s level of independence in daily activities, while the BBS and SARA scores specifically measure balance and ataxia, respectively. Improvements in these scores are indicative of reduced fall risk and enhanced motor coordination.

Assessment Item	At admission	3 weeks after rehabilitation	At discharge (Day 41)
FIM	Total: 78	Total: 121	Total: 121
	- Motor: 45	- Motor: 88	- Motor: 88
	- Cognitive: 33	- Cognitive: 33	- Cognitive: 33
BBS	22 (High fall risk)	53 (Significant reduction in fall risk)	56
SARA	20 (Severe ataxia)	3 (Substantial improvement)	3 (Stable)
Muscle Testing	- Upper extremities: Grip strength: Right 23.1 kg, Left 25.1 kg (MMT 5/5)	Not reported	- Upper extremities: Grip strength: Right 28.8 kg, Left 29.8 kg (MMT 5/5)
	- Lower extremities: Mild weakness bilaterally (MMT 4/5)	Not reported	- Lower extremities: Normal (MMT 5/5)
Cognitive Function	- MMSE: 30	Not reported	Not reported
	- TMT-B: 98 seconds		
	- FAB: 17 (Within normal limits)		
Diplopia	Present (impairing visual tasks and safe ambulation)	Present, but no longer significantly interferes with daily activities	Not reported
TUG Test	Not reported	Not reported	8.7 seconds
10-Meter Walk Test	Not reported	Not reported	5.5 seconds
6-Minute Walk Distance	Not reported	Not reported	525 meters

Rehabilitation program

An intensive, stepwise rehabilitation protocol was developed based on established programs for patients with ataxia [5]. During the first week, exercises concentrated on static and dynamic balance activities performed on a stable supportive surface. Given the patient’s severe ataxia and impaired consciousness, the initial focus on static and dynamic balance training was aimed at establishing a stable foundation for subsequent motor rehabilitation by enhancing core stability. Strengthening of trunk and limb control was achieved through a series of interventions, including standing exercises, postural holding exercises in quadrupedal positions, sitting and standing tasks, reaching exercises with slow, controlled movements, and progressive stepping tasks. Additionally, environmental modifications were implemented to mitigate the effects of diplopia, such as enhancing visual cues to facilitate the identification of target objects. For example, adjustments such as enhanced lighting and the placement of high-contrast visual cues were employed to improve visual orientation. Concurrently, ball-training exercises were incorporated to improve hand-eye coordination and reaction time, further supporting functional mobility and social engagement.

From the second week onward, the focus shifted to task-oriented practice emphasizing gait training. After achieving preliminary improvements in balance, gait training was introduced in the second week to facilitate the acquisition of functional ambulation skills, such as obstacle negotiation and stair climbing, thereby addressing residual ataxia and diplopia. The program incorporated applied stepping exercises, stair climbing, and obstacle negotiation to further improve postural stability. Due to persistent diplopia, oculomotor training was initiated. This oculomotor training aimed to expand the range of eye movements and enhance neural plasticity within the oculomotor pathways, thereby reducing the severity of diplopia. This component involved exercises designed to move the affected eye in the upward, downward, leftward, and rightward directions, as well as tasks to alternate focus between near and far objects.

After the third week, the patient began outdoor walking practice and engaged in activities aimed at promoting social reintegration. Tasks such as ambulating on uneven surfaces, using public transportation, and performing multitasking activities were introduced to simulate real-world scenarios. This phase was designed to transition the patient from controlled clinical exercises to real-world environments, thereby promoting social reintegration and preparing him for a return to work by simulating everyday activities. In preparation for returning to work, the patient also practiced operating computers, smartphones, and other devices used in his occupational setting. Furthermore, ball-training exercises were incorporated to help him achieve his personal goal of playing catch with his son (Table 2).

**Table 2:** Rehabilitation program Each intervention, ranging from static and dynamic balance exercises to oculomotor training, ws specifically targeted to address distinct deficits observed in BBE, such as impaired balance, diplopia, and coordination, thereby facilitating a comprehensive rehabilitation approach.

Period	Objectives/focus	Interventions/activities
Week 1	- Improve static and dynamic balance	- Static and dynamic balance exercises on a stable, supportive surface
	- Enhance trunk and limb control	- Standing exercises
	- Mitigate the effects of diplopia	- Postural holding exercises in quadrupedal positions
		- Sitting and standing tasks
		- Reaching exercises with slow, controlled movements
		- Progressive stepping tasks
		- Environmental modifications to enhance visual cues for target identification
Week 2 Onward	- Task-oriented practice with an emphasis on gait training	- Applied stepping exercises
	- Further improve postural stability	- Stair climbing
	- Address residual diplopia through oculomotor training	- Obstacle negotiation
		- Oculomotor training (exercises to move the affected eye upward, downward, leftward, and rightward, as well as tasks to alternate focus between near and far objects)
Week 3 Onward	- Adaptation to real-life scenarios via outdoor walking	- Outdoor walking practice
	- Promote social reintegration	- Walking on uneven surfaces
	- Prepare for return to work and personal goal achievement	- Using public transportation
		- Multitasking activities
		- Practicing the operation of computers, smartphones, and other devices used in the occupational setting
		- Ball-training exercises to work toward the personal goal of playing catch with his son

Follow-up and outcome

Within three weeks of initiating the rehabilitation program, the patient’s functional status improved markedly. For instance, the balance training initiated in week one likely contributed to the notable improvement in the BBS score from 22 to 53, while the reduction in diplopia following oculomotor training directly enhanced the patient’s safety during daily activities. The FIM score, which improved from 78 to 121, reflects a significant enhancement in the patient's ability to perform activities of daily living. This improvement, when compared to normative values, underscores the substantial recovery in functional independence. Although the patient continued to experience some difficulty with vision due to diplopia, targeted oculomotor training resulted in enhanced eye movement control, which significantly reduced the impact of diplopia on daily activities such as reading, using public transportation, and navigating crowded environments.

At discharge (41 days after admission), the patient’s balance and ambulation had further improved. The SARA score remained stable at 3, and the BBS score increased to 56 points. His performance on the Timed Up and Go (TUG) test improved to 8.7 seconds, and he completed the 10-meter walk test in 5.5 seconds, indicating a level of ambulatory independence suitable for outdoor walking. Moreover, his 6-minute walking distance increased to 525 meters, demonstrating enhanced endurance necessary for independent living and a return to work. Notably, his diplopia improved, enabling him to use public transportation, engage in outdoor activities without restrictions, and achieve his goal of playing catch with his son.

## Discussion

This case demonstrates that a structured and appropriately timed, individualized rehabilitation program can play a pivotal role in the recovery of a patient with BBE, a rare autoimmune disease. Although the patient underwent multiple immunotherapy interventions, he initially exhibited significant disability. However, comprehensive rehabilitation was associated with marked improvements in his FIM, BBS, and SARA scores, ultimately facilitating his return to society. It should be noted that this case report represents a single-subject study without a control group, which limits the generalizability of the findings. Additionally, potential subjective bias in the outcome measurements must be acknowledged.

BBE is characterized by oculomotor disturbances, ataxia, and both central and peripheral neurological deficits, primarily resulting from autoimmune-mediated inflammation of the brainstem [1]. While standard management strategies, such as corticosteroids, IVIg, and plasma exchange, effectively address the acute inflammatory process, residual functional deficits often persist [3]. As illustrated in this case, an individualized rehabilitation approach is critical for addressing these deficits.

The observed improvements in ataxia and oculomotor function may be attributed to the effectiveness of a task-specific rehabilitation approach, which aligns with current evidence in neurorehabilitation. Although the review by Kleim and Jones provides a general framework of neuroplasticity mechanisms, such as activity-dependent synaptic reorganization and enhanced connectivity within cortical and subcortical networks, its principles are particularly relevant in the context of Bickerstaff brainstem encephalitis (BBE) [6]. In BBE, inflammatory damage disrupts neural circuits governing motor coordination and oculomotor control. We hypothesize that task-specific training facilitated the re-establishment of these circuits through repeated, progressively challenging exercises, thereby contributing to the recovery of the patient's ataxia and oculomotor function. Further disease-specific investigations are warranted to elucidate these mechanisms in BBE.

Re-education of balance function is an essential component of rehabilitation aimed at improving postural control and ADLs. In this case, the patient may have benefited from interventions that promoted the realignment of vestibular and proprioceptive systems. Balance training is known to enhance sensory integration and compensate for vestibular deficits, while proprioceptive-focused exercises may augment somatosensory input and optimize sensorimotor integration [7]. These mechanisms likely underpinned the improvements observed in both ataxia and overall balance function [8].

While our case report demonstrated significant functional gains following a task-oriented rehabilitation program, it did not include objective neuroimaging data to confirm neuroplastic changes. Previous studies have reported that such interventions can enhance neuroplasticity by promoting activity-dependent synaptic reorganization [6]. In our study, neuroplasticity was inferred from marked improvements in clinical scales; nonetheless, the inclusion of imaging data in future research would provide more direct evidence of these underlying mechanisms.

Additionally, eye movement training may have contributed to the resolution of diplopia. Repetitive eye movement exercises probably enhanced plasticity within the oculomotor nuclei and their cortical projections, thereby facilitating the recovery of coordinated eye movements. This observation is consistent with previous studies showing that targeted visual and oculomotor exercises promote adaptive plasticity in the visual-vestibular pathway, which is crucial for restoring functional eye movement control [9].

Collectively, these findings underscore the importance of individualized, neurophysiologically informed rehabilitation strategies in managing complex neurological conditions such as BBE. Further research is warranted to elucidate the mechanisms underlying these improvements and to develop evidence-based rehabilitation protocols for patients with BBE and similar disorders.

## Conclusions

This case report shows that in a patient with BBE who presented with severe impaired consciousness and ataxia, an individualized and graded rehabilitation program implemented early after acute immunotherapy led to significant improvements in ADLs, balance, walking ability, and ataxia, ultimately facilitating reintegration into home life and work. Importantly, the patient’s personal goals, such as resuming playing catch with his son and returning to work, were achieved, underscoring that the observed numerical improvements translated into meaningful, real-world functional gains. These results underscore the critical role of appropriately timed, personalized rehabilitation in the recovery of BBE patients. Future studies should focus on establishing evidence-based treatment protocols by rigorously evaluating the effectiveness of rehabilitation interventions in similar patient populations.
